# Uncovering the antiinflammatory potential of *Lactiplantibacillus Plantarum* fermented *Cannabis Sativa L* seeds

**DOI:** 10.1038/s41538-024-00285-8

**Published:** 2024-06-28

**Authors:** LingYue Shan, Akanksha Tyagi, Hun-Ju Ham, Deog Hwan Oh

**Affiliations:** 1 Department of Food Science and Biotechnology, College of Agriculture and Life Sciences, Kangwon National University, Chuncheon, 200-701 Republic of South Korea; 2 Future F Biotech Co Ltd, Chuncheon, 24341 Republic of South Korea; 3 Department of Biological Environment, College of Agriculture and Life Sciences, Kangwon National University, Chuncheon, 24341 Republic of South Korea

**Keywords:** Secondary metabolism, Metabolomics

## Abstract

Inflammation acts as a dual role in disease initiation and progression, while *Cannabis sativa L*. (hemp) seeds, known for their abundance of anti-inflammatory phytochemicals, present a promising food source. Additionally, fermentation may optimize the food matrix, thereby augmenting its developmental prospects. This study explores the anti-inflammatory potential of hemp seeds fermented with 10 different probiotic strains. Among these, *Lactiplantibacillus plantarum* fermented hemp seeds (FHS) demonstrated a significant anti-inflammatory ability, accompanied by a reduction in the expression of critical inflammatory markers such as TLR4, NF-κBp65, and iNOS. Moreover, there is a noteworthy dose-dependent inhibition of inflammatory cytokines TNF-α, IL-6, IL-1β, and NO within a concentration range of 50 to 500 µg/mL. Subsequently, metabolomics analysis using UHPLC-QTOF-MS highlighted significant metabolic alterations in FHS compared to raw hemp seeds (RHS). Through multivariate, univariate, and correlation analyses, indolelactic acid (IA) and homovanillic acid (HVA) emerged as the main anti-inflammatory metabolites in FHS. Validation via HPLC confirmed the concentration of IA and HVA in RHS and FHS and both organic acids demonstrated lower IC_50_ values for TNF-α, IL-1β, IL-6, IL-18, and NO inhibition, showcasing their potent anti-inflammatory abilities. Furthermore, in vitro gastro-intestinal digestion coupled with the Caco-2 cell monolayer model validates the uptake and bioaccessibility of FHS, further affirming IA and HVA as major anti-inflammatory compounds. Overall, this research sets the stage for the development of novel hemp seed-based products targeting inflammation-associated disorders.

## Introduction

An inflammatory reaction occurs when the body’s immune system responds to harmful external stimuli, characterized by the overproduction of inflammatory factors, which in turn can contribute to the development of inflammatory diseases^1,2^. These diseases significantly burden the global population and are closely linked to morbidity and mortality. Initially, inflammation was revealed to correlate with homeostasis alterations in tissue; however, inflammatory signaling at the cellular level is recognized as a major actor in this complex phenomenon nowadays^1^. In-depth, effector T and B cells stimulate the production of pro-inflammatory cytokines, mainly including tumour necrosis factor-α (TNF-α), interleukin 6 (IL-6), interleukin 1 (IL-1) and interleukin 18 (IL-18), followed by triggering upstream or downstream protein expression and causing damage to the body^3^. Another inflammatory mediator, nitric oxide (NO), also interacts with immune cells to amplify the inflammatory response^4^. Currently, the primary drugs used to alleviate inflammatory reactions are biological agents such as anti-TNF-α (Humira), anti-IL-1 (Anakinra), and anti-IL-6 (Tocilizumab)^5^. But the application conditions of these biological agents are complicated. Therefore, more effective and safer natural bioactive compounds are needed to be explored from valuable food sources to provide greater selectivity in preventing inflammation.

*Cannabis sativa L*. (hemp) seeds have recently gained global recognition as a potentially valuable food source due to their rich protein, oil necessary to meet human dietary demands. Notably, hemp seeds contain low levels of psychoactive compounds such as delta 9-tetrahydrocannabinol (Δ9-THC), which enables their use as a food ingredient. Additionally, hemp seeds contain cannabinoic acids and their decarboxylated forms, such as cannabidiolic acid (CBDA), cannabigerolic acid (CBGA), cannabidiol (CBD), and cannabigerol (CBG), which hold significant nonpsychoactive pharmaceutical potential. These compounds have been reported to have significant anti-inflammatory, antioxidant, neuro-protective, and anxiolytic properties^6^. Moreover, hemp seeds harbour other bioactive compounds like phenolics, organic acids, unsaturated fatty acids and protein hydrolysates, which have demonstrated their ability to alleviate inflammatory conditions^6,7^. Despite significant efforts to uncover the physiological benefits of hemp seed as a food, limited research has been published due to its historical usage as an industrial product.

Fermentation technology is widely used in both conventional food products and the field of medicine. As a novel processing method, it is known as efficient for releasing various bioactive compounds, such as organic acids and peptides, from the cellular structure of food matrices^8^. Within the array of strains commonly employed in fermentation processes, lactic acid bacteria (LABs) stand out for their well-established safety profile and various bioactive properties. These include immunomodulatory, anti-inflammatory, anti-diabetic, and antioxidant effects^9^. Nevertheless, the present research exploring the utilization of LABs in fermenting hemp seed products with anti-inflammation remains relatively scant.

In biological systems, metabolomics is a highly efficient approach for quantifying numerous endogenous metabolites and identifying metabolic phenotypes^10^. By analyzing qualitative and quantitative metabolite data, researchers gain clearer insights into metabolic networks and speculate on metabolite functions^10^. As we know, liquid chromatography-mass spectrometry (LC-MS) is a powerful analytical tool that can comprehensively overview microbial metabolism in untargeted metabolomics. During fermentation, microbial cultures exhibit heightened metabolic activity, leading to an increase in the concentration of bioactive compounds. To date, there is a scarcity of information regarding the key metabolic pathways and differential metabolites of both raw and fermented hemp seeds. So identification of differential metabolites in hemp seeds during fermentation could be used to determine the anti-inflammation-related compounds and contribute to understanding the potential functionality of hemp seeds products.

This study aimed to assess the anti-inflammatory ability of *L. plantarum* fermented hemp seeds (FHS) and investigate the influence of *L. plantarum* on the alteration of hemp seed metabolites through untargeted metabolomics. Western blotting technology was employed to explore the TLR4/NF-κB-dependent signaling pathway by which FHS relieve inflammatory responses. The study involved using UHPLC-QTOF-MS to identify the overall metabolic profiles in raw hemp seeds (RHS) and FHS. Through multivariate, univariate, and correlation analysis, the primary potential metabolites associated with anti-inflammatory capability in the FHS were confirmed. To further validate effective anti-inflammatory metabolites in the FHS, we utilized a combination of chromatographic method to identify the presence and activity of the selected main anti-inflammatory metabolites in the FHS. Finally, uptake and bioaccessibility of FHS and two main anti-inflammatory compounds indolelactic acid (IA) and homovanillic acid (HVA) in FHS were evaluated using in vitro gastro-intestinal digestion coupled with the Caco-2 cell monolayer model. Nevertheless, this study was conducted at the cellular level, necessitating future clinical trials or in vivo research for potential human applications.

## Results and discussion

### The anti-inflammatory effects of RHS and FHS in the LPS-induced RAW 264.7 cell

In the inflammatory response, the initial explanation for the dominance of TNF in synovial systems relied on a linear model, suggesting that TNF would sequentially drive downstream cytokines, such as IL-1 and IL-6^11^. This recognition led to the successful treatment of autoimmune inflammatory disorders through TNF blockade^12^. Furthermore, clinical data indicate that inhibiting IL-1 and IL-18 shows therapeutic potential^11^. Another critical factor in the inflammatory reaction is NO, which acts as a transmembrane molecular signal and can cause damage to surrounding tissues^13^. As a result, targeting key mediators such as TNF-α, IL-6, IL-1β, and NO becomes crucial for effectively treating inflammatory diseases. These mediators were utilized to evaluate the anti-inflammatory effects of the samples initially.

**Fig. 1:**
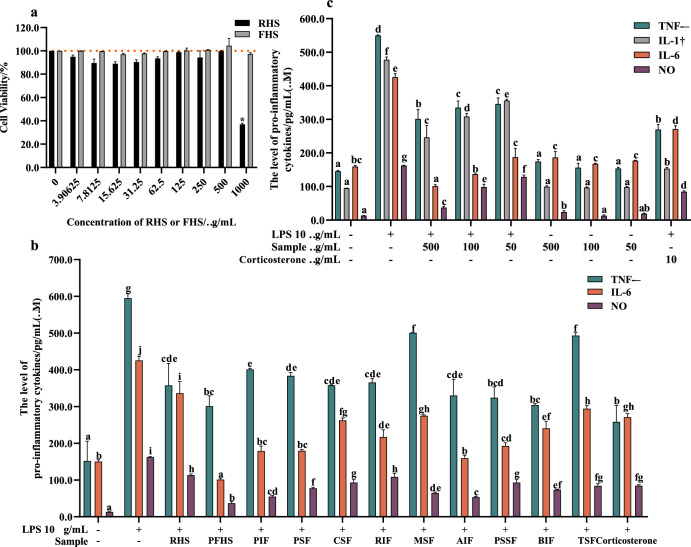
Anti-inflammatory capability of raw and fermented hemp seeds. **a** Effect of RHS and FHS on RAW 264.7 cells cytotoxicity. **b** Effect of fermented hemp seeds using 10 different *Lactic acid bacteria* on inhibiting TNF-α, IL-6, and NO levels. **c** Dose-dependent effect of FHS on inhibiting TNF-α, IL-1β, IL-6, and NO levels in inflammatory cells and the impact of various concentrations of FHS on TNF-α, IL-1β, IL-6, and NO levels in normal cells. In Fig. 1a–c, RHS raw hemp seeds, FHS *L. plantarum* fermented hemp seeds, PIF *L. Paracasei* fermented hemp seeds, PSF *L. Pentosus* fermented hemp seeds, CSF *L. Curvatus* fermented hemp seeds, RIF *L. Reuteri* fermented hemp seeds, MSF *L. Mesenteroides* fermented hemp seeds, AIF *P. Acidilactici* fermented hemp seeds, PSSF *P. Pentosaceus* fermented hemp seeds, BIF *S. Boulardii* fermented hemp seeds, TSF *S. Thermophiles* fermented hemp seeds. Corticosterone acetate was used as a positive drug. In these figures, “+” represents it contains the reagent, and “−” represents it does not contain the reagent. All values are expressed as the mean ± SD (*n* = 3). Different lowercase letters represent a significant difference (*p* < 0.05).

In our study, Fig. 1a showed that hemp seeds have no toxicity in raw 264.7 cells when used at concentrations less than 1000 μg/mL, making them suitable for further evaluation. We selected 10 strains, namely *Lactiplantibacillus plantarum subsp. plantarum*, *Lactobaciilus paracasei*, *Lactobacillus pentosus*, *Lactobacillus curvatus*, *Lactobacillus Reuteri*, *Leuconostoc mesenteroides*, *Pediococcus acidilactici*, *Pediococcus pentosaceus*, *Saccharomyces boulardii*, *Streptococus thermophiles* to individually ferment hemp seeds. The final pH values of the fermented hemp seeds ranged from 4.0 ± 0.5 to 4.6 ± 0.2, indicating that all strains were able to grow well in hemp seeds. To assess the anti-inflammatory activity of the ten different fermented hemp seeds at 500 μg/mL, we assayed TNF-α, IL-6, and NO inhibitory abilities, as shown in Fig. 1b. We found that cells treated with LPS provided an appropriate inflammatory model to assess hemp’s anti-inflammatory effects (Fig. 1b). The results showed that levels of TNF-α, IL-6, and NO in the RHS group are 357.5 pg/mL, 336.1 pg/mL, and 113.31 μM, respectively. Comparatively, Fig. 1b demonstrated that the FHS and BIF groups had significantly lower levels of TNF-α (301.25 pg/mL and 304.3 pg/mL, respectively) than the RHS group. Similarly, analysis of IL-6 levels confirmed that FHS (101.167 pg/mL) exhibited a significantly lower level compared to the RHS group (Fig. 1b). Figure 1b revealed that the NO inhibitory activity of FHS (37.17 μM) was remarkably improved compared to the RHS group. Based on these findings, we concluded that *L. plantarum* fermentation significantly enhances the anti-inflammatory activity of hemp seeds.

Among the 10 LAB strains used in fermentation, FHS showed superior anti-inflammatory activity. Furthermore, there was no cytotoxicity up to 1000 µg/mL concentration as depicted in Fig. 1a. When LPS-stimulated cells were treated with FHS, the levels of TNF-α, IL-6, IL-1β, and NO decreased in a dose-dependent manner (50 µg/mL to 500 µg/mL) (Fig. 1c). Notably, Fig. 1c indicated that there was no significant difference in the quantity of TNF-α between the 500 µg/mL FHS group and the positive drug group. To determine the inflammatory toxicity of FHS, the normal cells were exposed to varying concentrations of FHS. As seen from Fig. 1c, no significant alterations were observed in the levels of pro-inflammatory cytokines within the cells even at FHS doses as high as 500 μg/mL, compared to untreated cells.

These results showed the potential anti-inflammatory ability of RHS, attributed to the presence of anti-inflammatory compounds like CBD in the seeds^14^. After fermentation, the activity was significantly improved, consistent with findings from previous studies^15,16^. Further investigations involving *Lactiplantibacillus plantarum subsp. plantarum* and *Saccharomyces cerevisiae* treatment revealed that the fermented sample exhibited enhanced anti-inflammatory activity compared to the unfermented sample; this effect was may attributed to the down-regulation of the NF-κB pathway, resulting in nitric-oxide synthase (iNOS) inhibition, offering partial molecular insights into the anti-inflammatory properties^16^. From these findings, we can infer that FHS not only exerts a significant inhibitory effect on pro-inflammatory cytokines and mediators but also influences protein expression within the signaling pathway. In addition, researchers have recognized that LABs exhibit immune-enhancing effects and have the capability to generate metabolites with anti-inflammatory properties^17^. This discovery may provide a key explanation for the increased bioactivity of RHS after fermentation. Consequently, further investigations are needed to confirm the specific changes in metabolites produced during fermentation and their potential role in the enhanced anti-inflammatory effects.

### Effects of FHS on protein expression in the LPS-induced RAW 264.7 cell

**Fig. 2:**
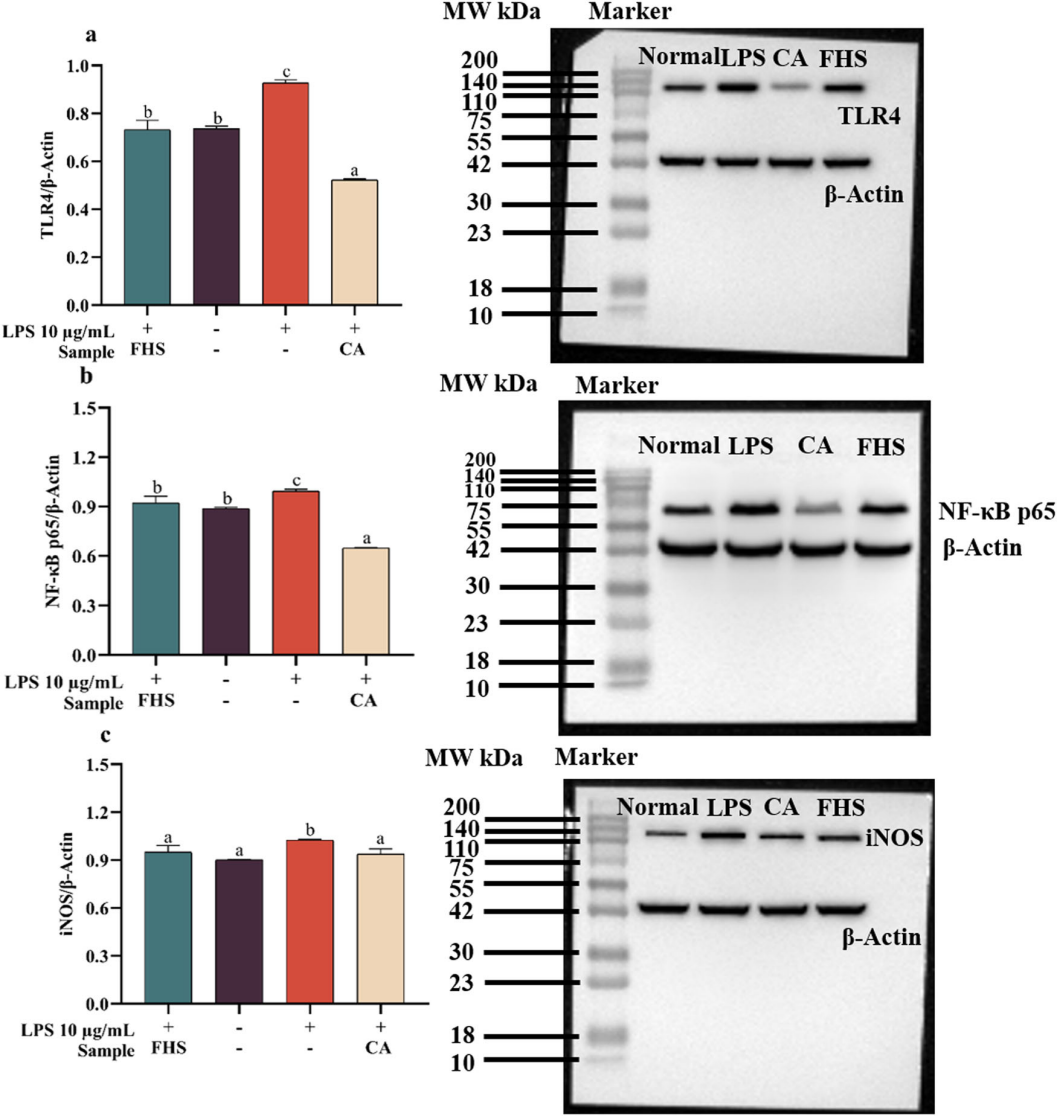
Effect of FHS (*L. plantarum* fermented hemp seeds) on TLR4/NF-κB signaling pathway in RAW 264.7 cells. **a** Representative western blots showing changes in the TLR4 expression and the intensities of bands were quantified using Image J software. **b** NF-κB p65 levels. **c** iNOS levels. In these figures, CA represents corticosterone acetate. All data are presented as the mean ± SD (*n* = 2). Different lowercase letters represent a significant difference (*p* < 0.05).

To investigate the anti-inflammatory mechanisms of FHS, we conducted a western blot technology to assess the protein expression levels in cells treated with FHS. As depicted in Fig. 2a, b, LPS stimulation led to increased inflammation and up-regulated expression of TLR4 and NF-κB p65. Both FHS and corticosterone acetate (positive drug) intervention significantly reduced the expressions of TLR4 and NF-κB p65 compared to the LPS-stimulated group. There was no significant difference between the FHS group and the normal cell group, suggesting that FHS did not induce any adverse effects under normal conditions. Under the inflammatory conditions, activated macrophages produce iNOS located downstream of the TLR4/NF-κB pathway, resulting in the generation of massive amounts of NO^16^. Therefore, we also investigated the effect of FHS on iNOS expressions. In Fig. 2c, the results indicated that both FHS and drug interventions effectively down-regulated the expression of iNOS in cells upon LPS stimulation. To sum up, FHS significantly reduced the inflammatory response by inhibiting the TLR4/NF-κB pathway in the LPS-stimulated Raw 264.7 cells.

In the inflammatory response progression, macrophage activation and elevated interleukin levels are commonly observed at the cellular level^12^. Generally, the stimulator LPS could activate the inflammatory reaction by binding to the TLR4 receptor, resulting in the activation of pro-inflammatory cytokines such as IL-6, TNF-α, and enzymes like iNOS^18^. The NF-κB signaling pathway is then established in the inflammatory response based on TLR4 receptor activation^18^. Our results demonstrated that the treatment of FHS effectively protects macrophages by reducing the levels of pro-inflammatory cytokines and mediators, including TNF-α, IL-1β, IL-6, IL-18, and NO (Fig. 1). Further investigation revealed that one of the mechanisms through which FHS combat inflammatory damage is by modulating the TLR4/NF-κB pathway.

### Multivariate analysis of UHPLC-Q-TOF/MS-based untargeted metabolomics

**Fig. 3:**
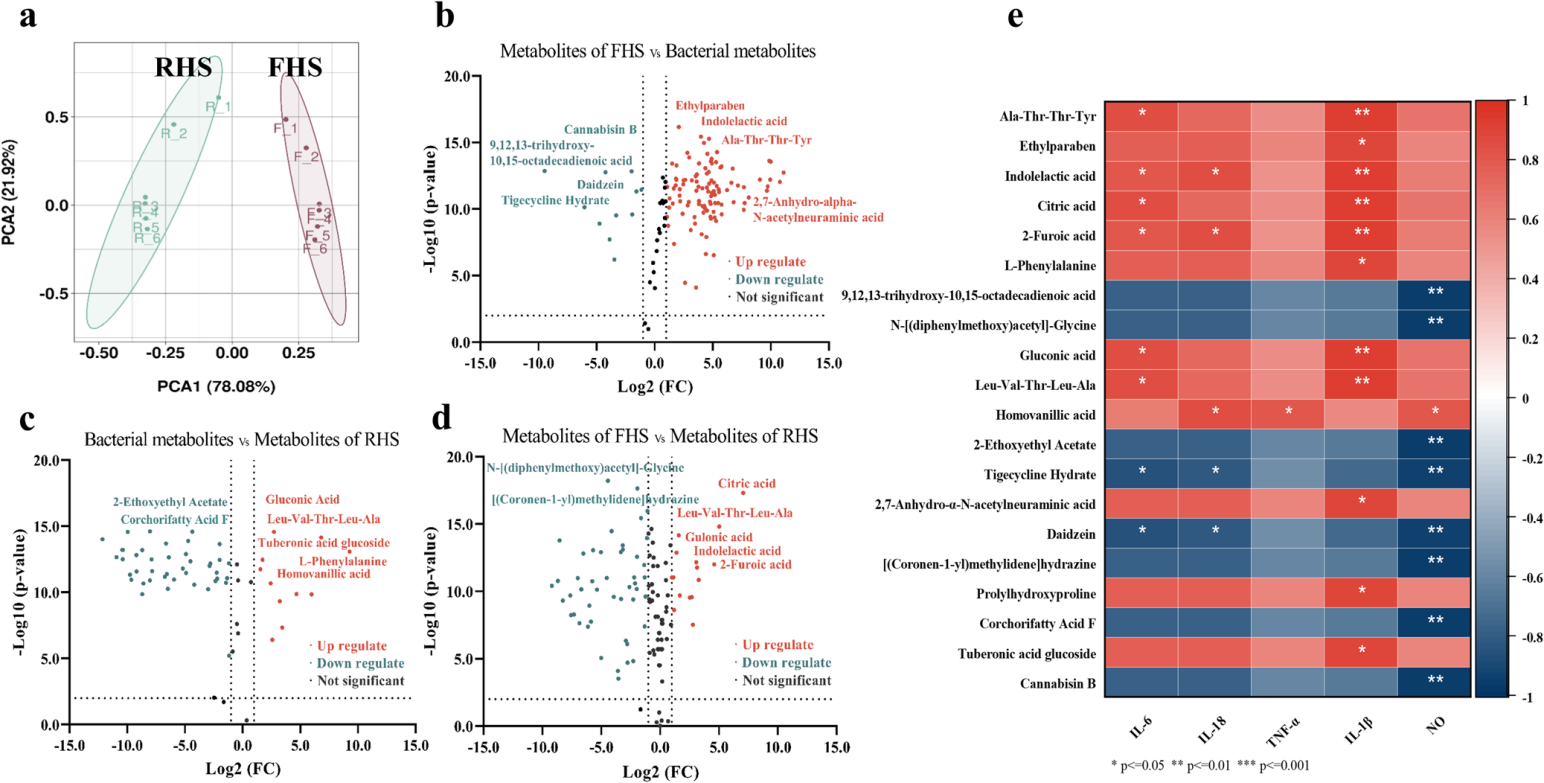
Multivariate, univariate, and correlation analysis of metabolites in RHS (raw hemp seeds), FHS (*L. plantarum* fermented hemp seeds), and bacterial (*L. plantarum* culture medium) groups. **a** Principal component analysis (PCA) score plot between RHS and FHS. **b** Volcano plot of differential metabolites between FHS and bacterial groups. **c** Volcano plot of differential metabolites between RHS and bacterial groups. **d** Volcano plot of differential metabolites between FHS and RHS groups. **e** Correlation map between differential metabolites and anti-inflammatory activity (IL-6, TNF-α, IL-18, IL-1β, NO). The color indicates the correlation coefficient and the star markers indicate a statistical difference. The orange color represents a positive correlation, and the blue color represents a negative correlation.

As an emerging method, untargeted metabolomics has been used to obtain high-through data and conduct batch correlation analysis based on the complete profiling of metabolites in food materials^13^. Our above results indicated that a significant improvement in the anti-inflammatory effect of hemp seeds after fermentation. Naturally, such enhancement in biological activity is likely accompanied by changes in various metabolites during the fermentation process. Hence, to gain deeper insights, we subjected the metabolomics data to multivariate statistics, aiming to elucidate the specific metabolite changes during fermentation and establish their correlation with the anti-inflammatory properties. The analysis of metabolomics data not only allows us to comprehend the fermentation-induced metabolic profile changes but also facilitates the identification of important potential inflammation-related compounds present in FHS. As a result of significant alterations of these compounds during fermentation, FHS demonstrates a substantial capacity to inhibit the expression of TLR4 and NF-κB p65 (Fig. 2). Supplementary Table 1 listed the changes in overall metabolic compounds in FHS and RHS groups, with a total of 107 identified metabolites and their relative content shown for both groups. Upon preliminary comparison, we observed a significant increase in several organic acid compounds after fermentation, including 2-furoic acid, gluconic acid, indolelactic acid, citric acid, and homovanillic acid. Previous studies have reported the beneficial effects of these compounds in suppressing inflammatory responses by inhibiting NF-κB signaling pathway activation, resulting in the reduced production of mediators and pro-inflammatory cytokines in RAW 264.7 cells^19–22^. Notably, indolelactic acid has been closely associated with an inflammatory biomarker^23^. The PCA result (Fig. 3a) indicated a significant separation between the metabolic profiles of RHS and FHS in the two principal components, signifying distinct differences in the metabolites they contain. These findings highlight the substantial impact of fermentation on the metabolic composition of hemp seeds.

### Univariate analysis of UHPLC-Q-TOF/MS-based untargeted metabolomics

To examine the detailed differences in metabolite profiles between groups, the univariate analysis was employed and the results were visualized in volcano plots (Fig. 3b–d). In these plots, up-regulated metabolites are represented by orange dots, while down-regulated metabolites are depicted by blue dots. Specifically, we screened the 20 most important differential metabolites for further correlation analysis based on a comparison of *q*-value and fold change value between groups. These metabolites include Ala-Thr-Thr-Tyr, ethylparaben, indolelactic acid, citric acid, 2-furoic acid, L-phenylalanine, 9,12,13-trihydroxy-10,15-octadecadienoic acid, N-[(diphenylmethoxy)acetyl]-glycine, gluconic acid, Leu-Val-Thr-Leu-Ala, homovanillic acid, 2-ethoxyethyl acetate, tigecycline hydrate, 2,7-Anhydro-α-N-acetylneuraminic acid, daidzein, [(Coronen-1-yl) methylidene] hydrazine, prolylhydroxyproline, corchorifatty acid F, tuberonic acid glucoside, cannabisin B (Fig. 3b–d). The detailed fragment ions, retention time, and relative content of these compounds are listed in Table 1. These 20 differential metabolites play a crucial role in revealing the metabolic changes that occurred during fermentation and may be key contributors to the anti-inflammatory effects of FHS.

**Table 1:** List of tentatively identified compounds with significant changes in relative content in FHS and RHS using HPLC-ESI-QTOF-MS/MS

No.	Formula	Compound Name	Retention Time/min	m/z [M-H]^-^	Area (FHS)	Area (RHS)
1	C_9_H_11_NO_2_	L-Phenylalanine	2.47	164.072	1500	170,000
2	C_5_H_4_O_3_	2-Furoic Acid	1.27	111.009	780,000	-
3	C_11_H_17_NO_8_	2,7-Anhydro-α-N-acetylneuraminic Acid	1.34	290.0886	300,000	-
4	C_10_H_16_N_2_O_4_	Prolylhydroxyproline	5.62	227.1042	200,000	-
5	C_6_H_12_O_7_	Gluconic Acid	17.56	195.051	1,300,000	3900
6	C_11_H_11_NO_3_	Indolelactic Acid	15.9	204.0668	2,100,000	5500
7	C_6_H_8_O_7_	Citric Acid	3.28	191.0201	3,900,000	120,000
8	C_18_H_32_O_5_	Corchorifatty Acid F	20.07	327.2177	16,000	1,400,000
9	C_15_H_10_O_4_	Daidzein	18.58	253.051	-	690,000
10	C_18_H_32_O_5_	9,12,13-trihydroxy-10,15-octadecadienoic acid	17.02	328.0211	1100	410,000
11	C_34_H_32_N_2_O_8_	Cannabisin B	18.86	595.2082	5700	2,900,000
12	C_20_H_30_N_4_O_8_	Ala-Thr-Thr-Tyr	10.22	453.1995	260,000	-
13	C_24_H_45_N_5_O_7_	Leu-Val-Thr-Leu-Ala	6.77	286.1772	42,000	-
14	C_17_H_17_NO_4_	N-[(diphenylmethoxy)acetyl]-glycine	1.28	176.9361	43,000	210,000
15	C_25_H_14_N_2_	[(Coronen-1-yl) methylidene] Hydrazine	1.06	341.1092	120,000	16,000,000
16	C_18_H_28_O_9_	Tuberonic Acid Glucoside	13.87	387.1666	230,000	-
17	C_9_H_10_O_4_	Homovanillic acid	13.05	301.1089	220,000	7600
18	C_6_H_12_O_3_	2-Ethoxyethyl Acetate	11.12	131.0716	4200	320,000
19	C_9_H_10_O_3_	Ethylparaben	14.34	165.0559	33,000	-
20	C_29_H_41_N_5_O_9_	Tigecycline Hydrate	11.48	602.2831		2200

Based on these results, the highest proportion of compounds in the FHS belonged to organic acids, alkaloids, and derivatives, followed by amino acids, and peptides. As fermentation progressed, the microbial cells underwent autolysis leading to release of cellular exudates, thus the organic acids, alkaloids, and derivatives compounds pronounced changes^24^. This observation aligns with previous literature documenting notable alterations in organic acid abundance during the fermentation of hemp seed bran^25^. Regarding amino acids and peptides, they were attributable to the hydrolysis of proteins in the raw material and bacterial autolysis^26^. In addition, amines can be derived from amino acids through decarboxylation reactions, producing alkaloids that constitute approximately 20% of plant-based secondary metabolites^27^. Alkaloids are renowned for their high biological activities and have gained importance in medicine. Thus, the changes observed in amino acids and the subsequent formation of alkaloid compounds during fermentation might account for the observed above phenomenon. In conclusion, fermentation can enhance the utilization value of the food matrix by bringing about changes in these differential metabolites. Furthermore, the alterations in flavor-related metabolites, such as amino acids and peptides during fermentation, hold crucial significance in the development of novel functional food products.

### Correlations of differential metabolites with the anti-inflammatory activities

Spearman’s correlation analysis was conducted to investigate the relationship between differential metabolites in hemp seeds during fermentation and their contributions to anti-inflammatory ability. In this analysis, the 20 differential metabolites were selected as the variables and their correlated relationship is depicted in Fig. 3e. Among these 20 differential metabolites, 7 potential compounds were found to exhibit a significant positive association with anti-inflammation. These compounds primarily belong to organic acids and peptides. Specifically, the inhibitory activity of IL-6, IL-18, TNF-α, IL-1β, and NO production strongly and positively correlated with Ala-Thr-Thr-Tyr, indolelactic acid, citric acid, 2-furoic acid, gluconic acid, Leu-Val-Thr-Leu-Ala, homovanillic acid, most of which are classified as organic acid compounds (Fig. 3e). Other studies have proved that organic acid compounds can be as major contributors to anti-inflammatory activity. These components have demonstrated their effectiveness in reducing LPS-induced inflammation, and their potential mechanism of action may involve the TLR4/IKK/NF-kB signaling pathway^28^. Interestingly, our study showed that anti-inflammatory peptides emerged in hemp seeds after fermentation. These peptides likely bind with factors involved in inflammatory pathways, such as TLRs and NF-κB, thus reducing the inflammatory response^29^. However, it is essential to note that peptides are unstable. Therefore, we need to conduct more detailed investigations to thoroughly validate the potency and molecular stability of these peptides present in FHS.

### Confirmation of anti-inflammatory organic acids compounds in FHS

**Fig. 4:**
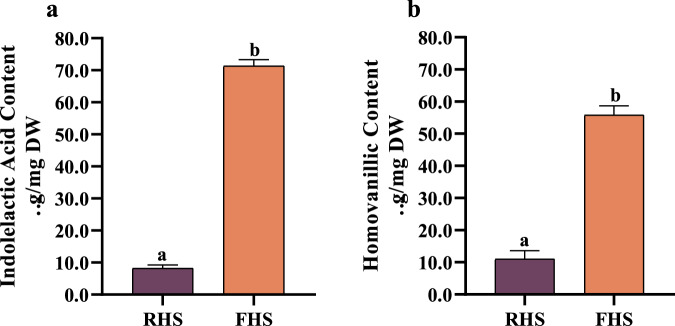
Quantitative analysis of indolelactic acid (IA) and homovanillic acid (HVA). **a** The content of IA in RHS (raw hemp seeds) and FHS (*L. plantarum* fermented hemp seeds) groups. **b** The content of HVA in RHS and FHS groups. The data are presented as the mean ± SD (*n* = 3). Different lowercase letters represent a significant difference (*p* < 0.05).

Based on the results obtained from the multivariate, univariate, and correlation analysis presented above, we employed the chromatographic method using HPLC-DAD to identify anti-inflammation-related organic acid compounds present in FHS. By quantifying the strongly anti-inflammation-related compounds identified in the correlation analysis, we found that indolelactic acid (IA) and homovanillic acid (HVA) displayed the most significant changes during the fermentation and exhibited the highest content after fermentation (Fig. 4a, b). In detail, Fig. 4a, b illustrated that the RHS group contained 7.6 µg/mg of IA and 9.2 µg/mg of HVA. However, the content of IA and HVA remarkably increased to 72.7 µg/mg and 57.9 µg/mg after fermentation, respectively.

The significant increase of IA and HVA in FHS is highly likely to be the reason for the enhanced anti-inflammatory activity of FHS. Thus we separately examined the anti-inflammatory activity of these two compounds to better understand their individual contributions. Table 2. illustrated the half inhibition concentrations (IC_50_) of IA and HVA. Both compounds exhibited excellent anti-inflammatory ability. IA exhibited higher inhibitory activity against IL-1β, IL-6, and NO, with IC_50_ values of 9.11 ± 0.21 µg/mL, 10.67 ± 1.04 µg/mL, 8.74 ± 0.32 µg/mL, respectively. On the other hand, HVA displayed higher inhibitory activity against TNF-α and IL-18, with IC_50_ values of 11.84 ± 1.02 µg/mL and 10.94 ± 0.31 µg/mL, respectively. These findings suggested that both IA and HVA contribute significantly to the potent anti-inflammatory effects observed in FHS. These findings closely resemble those documented in previous literature, highlighting the significant potential of IA and HVA in biological activities, including antioxidant and anti-inflammatory effects^30,31^.

**Table 2:** IC_50_ values of IA and HVA anti-inflammatory activities

	IC_50_ (μg/mL)				
	TNF-α	IL-1β	IL-6	IL-18	NO
IA	12.63 ± 2.00^a^	9.11 ± 0.21^a^	10.67 ± 1.04^a^	12.03 ± 1.02^b^	8.74 ± 0.32^a^
HVA	11.84 ± 1.02^a^	10.21 ± 1.00^ab^	11.53 ± 1.62^a^	10.94 ± 0.31^ab^	9.19 ± 0.28^a^

### Changes in IA and HVA contents, and antiinflammatory capacity of FHS and RHS during digestion

**Fig. 5:**
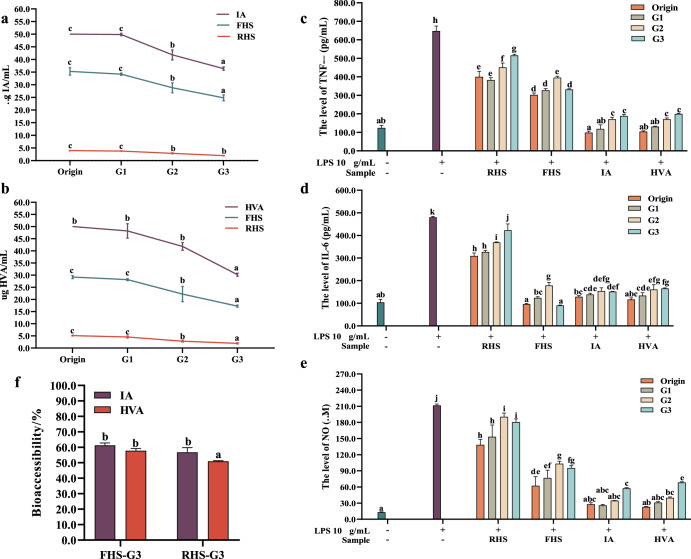
In vitro simulated digestive and bioaccessibilty assays. The changes of the content of IA (**a**) and HVA (**b**) in FHS and cellular anti-inflammatory activity of FHS, RHS, IA, and HVA (**c**–**e**) during digestion. **f** The bioaccessibilty of FHS and RHS. In figures, G1 means the saliva digestive group, GD2 means the gastric digestive group, and G3 means the intestinal digestive group. All data are presented as the mean ± SD (*n* = 3). Different letters indicate significant differences (*p* < 0.05).

In this study, hemp seeds exhibited notable anti-inflammatory properties following fermentation with *L. plantarum*, with IA and HVA identified as the primary metabolites responsible for this effect. Thus, IA and HVA act as pivotal detection indicators for assessing the bioavailability of FHS throughout the entire ingestion. We examine variations in their content and activity at each stage to evaluate FHS availability during digestion. As shown in Fig. 5, both IA and HVA, along with their levels within FHS, performed relatively stable throughout the simulated digestive process. The exposure of IA and HVA in the simulated oral cavity did not compromise their stability, as the environment had no detrimental impact on their integrity (Fig. 5a, b). This alignment with our previous HPLC validation findings provides additional evidence of IA and HVA stability, even in the presence of α-amylase. During simulated gastric and intestinal digestion, we observed a minor reduction in IA content within FHS, although IA itself exhibited no degradation (Fig. 5a). As a small molecule lipophilic tryptophan metabolite generated by gut microbiota^32^, IA lacks amide bonds, rendering it stable in simulated gastric and intestinal fluids and conducive to utilization. While reports indicate the accumulation of millimolar indole compounds in the gastrointestinal tract^33^, studies specifically addressing the digestion of IA are limited. The slight decrease in IA content in FHS may be attributed to the involvement of tryptophan in the indole derivative pathway, which works together with enzymes to consume IA^32^. In comparison to IA, HVA within FHS showed relative stability in gastric juice but underwent a significant decrease in the alkaline environment of intestinal juice (Fig. 5b). This could be attributed to HVA’s susceptibility to digestive enzymes and pH variations, which may result in degradation or biotransformation^34^. An alternative explanation might involve interactions with other constituents of the FHS extract (e.g., amino acids and fatty acids), potentially leading to the formation of complexes that interfere with HVA quantification^34^.

The fluctuation in anti-inflammatory activity corresponds to changes in the content of IA and HVA, as depicted in Fig. 5c–e. The activity of IA and HVA remains unchanged after salivary digestion, whereas a significant increase is observed following gastric and intestinal digestion (Fig. 5c–e). This could be attributed to the primary action sites of IA and HVA, which are predominantly located in the gastric and intestinal mucosa^35^. Additionally, as illustrated in Fig. 5b, the increase in branching of the alkyl chain resulting from hydrolysis enhances the activity of HVA during the digestion process^35^. Interestingly, the bioactivity of HVA appears to be independent of its chain length, with 1-methylated compounds demonstrating greater activity^35^. Saliva, gastric, and intestinal samples of FHS exhibit notable anti-inflammatory activity, which significantly increases after gastric digestion but decreases somewhat after intestinal digestion. However, there is no significant difference compared to the original FHS, as indicated in Fig. 5c–e. The observed activity recovery could be related to the interaction of other components in FHS, such as fatty acids and phenolic compounds, which can be sensitively activated by the acidic and alkaline environment^34^.

### Bioaccessibility of IA and HVA in FHS and RHS after digestion

Bioaccessibility refers to the ease with which a substance can enter an organism and be utilized by it. Food material, it quantifies the amount or proportion of compounds released from the food matrix in the digestive tract that can be absorbed into the blood^36^. We assessed the bioaccessibility of our main active compounds, IA and HVA, using an in vitro simulated digestion and intestinal Caco-2 cell monolayer model. The Caco-2 cell model is recognized as an effective system for studying the bioavailability of whole-food phytochemicals by evaluating the transmission of main compounds^36,37^. As depicted in Fig. 5f, the bioaccessibility of IA and HVA was measured at 61.27 ± 1.04% and 57.79 ± 1.77%, respectively in FHS. In RHS, the bioaccessibility of IA and HVA was determined to be 56.825 ± 2.37% and 50.945 ± 1.29%, respectively. No significant difference in the bioaccessibility of active compounds was observed between the FHS and RHS. This suggests that when FHS is orally consumed, approximately 60% of anti-inflammatory compounds can be absorbed and utilized by the body.

## Methods

### Reagents and materials

Cheungsam *Cannabis sativa L*. (hemp) seeds were obtained from the Cha Hemp Industry Ltd., Chuncheon, Korea. The macrophage (RAW 264.7) cell line and Caco-2 cells were obtained from the American Type Culture Collection, Manassas, VA, USA. 10 LABs used in this study were isolated from Korean fermented food (kimchi) for fermentation. To ensure the safety of LABs as normal flora, kimchi products with a history of safe consumption were selected. All bacteria stock cultures were stored at −80 °C.

Dulbecco’s Modified Eagle’s Medium (DMEM), fetal bovine serum (FBS), 0.05% Trypsin-EDTA (1X), and penicillin were purchased from Thermo Fisher Scientific, USA. Lipopolysaccharide (LPS) from *Escherichia coli* O111:B4, corticosterone acetate, RIPA buffer, homovanillic acid (HVA) > 99%, indolelactic acid (IA) > 99%, dimethyl sulfoxide (DMSO), α-amylase, pepsin, pancreatin, and bile salts mixture were purchased from Sigma Aldrich, USA. All primary and secondary antibodies were purchased from Cell Signaling Technologies, Danvers, MA, USA. EZ-Cytox enhanced cell viability assay kit was purchased from DoGenBio Co., Ltd, South Korea. HPLC grade methanol was obtained from Allpolab Co., Ltd, South Korea. All ELISA Kits used in this study were purchased from Thermo Fisher Scientific Solutions Co., Ltd, USA. All western blotting standards and chemicals were purchased from Bio-RAD, USA. Other chemical reagents used were analytical grade.

### Samples preparation

#### Fermentation procedure

The raw seeds were fermented following the previously described protocol with slight modifications^38^. Briefly, an electric mill (Hanil electric co., Ltd., South Korea) was used to pulverize the RHS into powder. The powder was mixed with distilled water at a 1:20 (W/V) ratio and subjected to sterilization by autoclaving at 121 °C for 15 min. Then the sterilized powder was inoculated with *Lactiplantibacillus plantarum subsp. plantarum* OHSH:1 at a concentration of 10% (v/v, 2 × 10^8^ CFU/mL). The fermentation process was carried on the 150 rpm rotary shaker for 24 h at 37 °C. The FHS was freeze-dried by Free-Drier (Labconco Corporation, USA) and stored at −20 °C until further studies.

### Extraction

The extracting followed the published procedure^39^, with minor adjustments. The RHS and freeze-dried FHS powder was extracted in 70% ethanol at a ratio of 1: 20 (W/V) for 24 h at room temperature in an electric shaker (RK-2D, DAIHAN scientific, Korea). Following extraction, the samples were centrifuged at 4000 × *g* for 10 min (Union 32 R plus, Hanil Science Industrial, Korea). The supernatant and precipitate were collected, respectively. The precipitate was then subjected to a second extraction by adding 70% ethanol in a ratio of 1:20 (W/V) at room temperature for 24 h. The supernatant and precipitate were collected again under the same centrifugal conditions as the first extraction. This process was repeated for a total of three times. The supernatant obtained from the three extractions and freeze-dried (Labconco Corporation, USA). The freeze-dried extract was stored at −20 °C until further analysis.

### Cell culture

RAW 264.7 cells were grown in DMEM containing 10% FBS and 1% penicillin. Cells were maintained at 37 °C in a humidified environment with a 5% CO_2_ incubator^40^. Sub-cultured cells were treated with 0.05% Trypsin-EDTA (1X)^40^.

### Cytotoxicity assay

The cytotoxicity of the extract dissolved in serum-free media was evaluated using the EZ-Cytox assay kit with slight modifications following the protocol^41^. RAW 264.7 cells were seeded at a density of 1 × 10^5^ per well in a 96-well plate. Then the cells were treated with extracts of different concentrations and incubated for 24 h. Subsequently, 10 μL EZ-Cytox was added and further incubated for 2 h. A SpectraMax i3 plate reader (Molecular Devices Korea, LLC, Korea) was used to read absorbance at 450 nm. Cell viability was calculated according to the instructions provided with kit.

### Anti-inflammatory assays

The effect on inhibiting inflammatory responses of macropahges was performed as previously reported with slightly modified^13^. The levels of TNF-α, IL-1β, IL-6, IL-18, and NO in the cell culture supernatant, were determined using ELISA kits as per the kit’s protocol. Briefly, the cells were seeded at a density of 1 × 10^5^ per well in 96-well plates and incubated for 12 h, followed by stimulation using 50 µL of 10 µg/mL LPS and incubated for 4 h. The 100 µL extract samples were then added to the wells and incubated for 24 h. As a positive control, 10 µg/mL corticosterone acetate dissolved in DMSO and then treated cells.

### Immunoblotting analysis

The whole cell lysate was obtained by adding RIPA buffer to the cells, and the mixture was centrifuged (Micro 12, Hanil Science Industrial, Korea) at 7711 × *g* for 5 min. A modified lowry protein assay kit (Thermo Scientific, USA) was used to evaluate the protein content of the supernatant. The supernatant with uniform protein content was subsequently subjected to Sodium Dodecyl Sulphate Polyacrylamide Gel Electrophoresis (SDS-PAGE) according to the protocol provided by Bio-RAD, USA. The target protein expression was incubated with primary antibodies at a 1:1000 dilution overnight at 4 °C. Subsequently, secondary antibodies with a dilution of 1:3000 were applied and incubated for 1.5 h at room temperature. An enhanced chemiluminescence kit and a chemiluminescence detector (Fusion FX, Vilber Laurmate, France) were utilized to visualize the gel. The data was normalized to the expression levels of the housekeeping protein β-actin.

### UHPLC-QTOF-MS identification

The metabolites were identified using UHPLC-QTOF-MS according to methodology with slight modifications^42^. Ultrahigh-pressure liquid chromatography (UHPLC) was equipped with an AD pump, AD autosampler, AD column oven, degasser, controller and photodiode array (PDA) detector (ExionLC), as well as a quadrupole time-of-flight mass spectrometer (QTOF-MS) (SCIEX ExionLC AD machine, MA, USA). Briefly, the extracts dissolved in 70% ethanol (5 mg/mL) and were filtered through 0.22 μm Millex syringe filters. Chromatographic separation was performed on Accucore column C18 (100 × 3 mm, Thermo Fisher Scientific, Waltham, MA, USA). The compounds were eluted using the mobile phase, including solvent A (water containing 0.1% formic acid) and solution B (100% methanol) at a 0.5 mL/min flow rate. The following linear gradient elution program was used: 0–4 min, 9–14% B; 4–5 min, 14–15% B; 5–7 min, 15% B; 7–9 min, 15–17% B; 9–10 min, 17–19% B; 10–11 min, 19% B; 11–13 min, 19–26% B; 13–14 min, 26–28% B; 14–15 min, 28–35% B; 15–17 min, 35–40% B; 17–18 min, 40–48% B; 18–19 min, 48–53% B; 19–23 min, 53–70% B; 23–24 min, 70–9% B; and 24–30 min, 9% B. The mass spectrometry (SCIEX, Framingham, MA, USA) was working under positive (ESI+) and negative (ESI−) electrospray ionization modes. The source temperature was maintained at 320 °C. The spray and tube lens voltage was controlled at 120 V (3.6 kV) and −120 V (2.7 kV) for the positive and negative ion modes, respectively. The sheath gas flow and units and an aux gas flow were controlled at 40 and 8 arb, respectively. The mass scan range was from 115 to 1000 m/z, and the scanning time was set for 1 s.

### HPLC-DAD analysis

The procedure was conducted based on a previous study with modifications^43^. Briefly, the standards and samples were dissolved in ddH_2_O. All the standards and samples were prepared in 70% ethanol and then filtered through 0.22 μm Millex syringe filters. HPLC analysis was performed using Agilent 1260 series system equipped with a quaternary gradient pump and a DAD detector. Separation was achieved using a Phenomenex Gemini C18 column (4.6 × 250 mm, 5 μm) from Agilent, USA. The mobile phase (MP) A consisted of 50 mM ammonium formate in water (adjusted to pH 6.55 with formic acid). MPB consisted of 50 mM ammonium formate in methanol (pH 6.55). The gradient was set as follows: 0–10 min with 0% MPB; 10–20 min with 40% MPB; 20–30 min with 100% MPB. The flow rate was set at 1 mL/min, and the injection volume was set to 10 μL.

### In-vitro simulated digestion and bioaccessibility

#### In-vitro simulated digestion

In-vitro simulated digestion was conducted following a previously established protocol with some modifications^37,44,45^. Throughout the entire digestive process, the pH value was adjusted using 6 M HCl and 0.9 M NaHCO_3_. The tested samples included 500 μg/mL FHS, 500 μg/mL RHS, 50 μg/mL IA, and 50 μg/mL HVA, respectively. Each sample was further divided into three groups (G1, G2, G3), with each group represented by three replicates. The simulated digestion began from the salivary step, wherein 0.25 mL saliva (containing 16.25 mg α-amylase in 12.5 mL of 1 mM CaCl_2_ at pH 7.0) was added to each group and incubated for 10 min. G1 was subsequently collected and reserved as saliva samples. For gastric digestion, the pH values of the remaining two groups were adjusted to 2.0, and 0.25 mL gastric juice (containing 0.4 g pepsin in 10 mL 0.1 M HCl) was added to each group, followed by incubation for 2 h. G2 was collected, and its pH was adjusted to 7.0, designating it as gastric samples. Next, the pH value of G3 was adjusted to 7.5, and 2.5 mL intestinal juice (containing 1 g pancreatin, and 6 g bile salts in 50 ml 0.1 M NaHCO_3_) was added to the G3 to initiate intestinal digestion. After 2 h, G3 was reserved as intestinal samples. All samples were filtered and immediately utilized for further analysis.

### Bioaccessibility of bioactive compounds in Caco-2 cellular model

The bioaccessibility of the samples was performed according to the published protocol^36,37^ with minor adjustments. After simulated digestion, intestinal samples from FHS and RHS were marked as FHS-G3 and RHS-G3 and were taken out for bioaccessibility analysis. In this study, the IA and HVA were confirmed as the primary anti-inflammatory compounds during digestion. Hence, the in vitro bioaccessibility (%) was calculated by the ratio of IA and HVA mass in the bioaccessible fraction to the total mass based on the Caco-2 model. Caco-2 cells were cultured at 37 °C in DMEM supplemented with 10% FBS and 1% penicillin-streptomycin. During the measured process, the cells at a density of 1 × 10^6^ cells/ml were seeded on 24-well Transwell plates, and incubated continuously for 14 days to form monolayers. Following washing with HBSS (pH 6.8), the monolayers were exposed to 2 mL of FHS-G3 and RHS-G3 for 2 h. After incubation, the upper liquid and the lower liquid were collected individually for HPLC analysis.

### Statistical analysis

Each experimental group was set to be *n* = 6 in metabolomics analysis, *n* = 3 in ELISA analysis or *n* = 2 in immunoblotting analysis. The results were described as average ± standard deviation (SD). The data were analyzed using GraphPad Prism 8.0 (GraphPad Software, La Jolla, CA, USA) and one-way ANOVA at *p* < 0.05 significance stage using the SPSS Statistics 24.0 (GraphPad Software, La Jolla, CA, USA). The metabolomics results were performed using the OmicStudio tools at https://www.omicstudio.cn/home. The results of immunoblotting were quantified by NIH ImageJ software (National Institutes of Health, Bethesda, MD, USA).

### Supplementary information

Supplementary Information

## Data Availability

Data will be made available on request.
