# Supplementary material for: Uncovering the antiinflammatory potential of Lactiplantibacillus Plantarum fermented Cannabis Sativa L seeds
Source: NPJ Sci Food. 2024 Jun 28;8:42. doi: 10.1038/s41538-024-00285-8 (PMC11214619; doi:10.1038/s41538-024-00285-8)
Supplement: Supplementary file 1 — Supplementary Information [file 41538_2024_285_MOESM1_ESM.pdf]

Supplementary Table 1. List of all known tentatively identified compounds in FHS and RHS using HPLC-ESI-QTOF-MS/MS

**No.**	**Formula**	**Compound Name**	**Retention Time/min**	**m/z [M-H]$^-$**	**Area (FHS)**	**Area (RHS)**	**Area** (Probiotic *L. plantarum*)
1	$C_9H_{12}N_2O_4$	Levoamine	2.05	211.0731	58000	-	100
2	$C_{17}H_{20}N_4O_6$	Fiboflavin	15.19	375.1307	4400	-	-
3	$C_{20}H_{15}NO_5$	Sanguinarine Pseudobas	19.52	348.0882	-	180000	-
4	$C_{13}H_{12}N_4O_2$	8-Phenyltheophylline	15.14	255.0892	-	12000	-
5	$C_8H_{16}OS_3$	Foetisulfide A	20.01	223.029	-	1500	-
6	$C_{33}H_{44}N_2O_3$	Dendrocrepin	16.67	515.3278	-	270000	720
7	$C_{17}H_{30}N_4O_5$	Ala-Leu-Ala-Pro	5.27	369.2145	19000	-	-
8	$C_{20}H_{30}N_4O_8$	Ala-Thr-Thr-Tyr	10.22	453.1995	260000	-	-
9	$C_{24}H_{45}N_5O_7$	Leu-Val-Thr-Leu-Ala	6.77	286.1772	42000	-	-
10	$C_{19}H_{30}N_4O_7$	Asp-Val-Pro-Pro	10.24	425.2045	25000	-	-
11	$C_{15}H_{25}N_3O_6$	Val-Glu-Pro	13.9	342.167	14000	-	-
12	$C_9H_{11}NO_2$	L-Phenylalanine	2.47	164.072	1500	170000	140
13	$C_4H_9NO_3$	L-Threonine	0.99	118.0514	-	19000	2000
14	$C_{15}H_{17}N_3O_5$	Asp-Trp	7.84	318.1098	-	1600000	-
15	$C_5H_8O_5$	3-Hydroxypentanedioic Acid	1.35	147.0299	430000	98000	500
16	$C_5H_7NO_3$	L-Pyroglutamic Acid	1.43	128.0357	580000	-	1000
17	$C_7H_{11}NO_5$	2-Acetamidopentanedioic Acid	1.62	188.0565	440000	19000	2300
18	$C_{10}H_{14}N_2O_6$	Ribosylimidazoleacetic Acid	1.51	257.0786	130000	1900	440
19	$C_4H_6O_4$	Succinic Acid	1.65	117.0196	340000	-	200
20	$C_5H_4O_3$	2-Furoic Acid	1.27	111.009	780000	-	3200
21	$C_{11}H_{17}NO_8$	2,7-Anhydro-α-N-acetylneuraminic Acid	1.34	290.0886	300000	-	-
22	$C_6H_8O_6$	Ascorbic Acid	1.75	175.0253	370000	6700	3300
23	$C_5H_6O_4$	Itaconic Acid	4.01	129.0196	170000	-	1000
24	$C_{10}H_{16}N_2O_4$	Prolylhydroxyproline	5.62	227.1042	200000	-	-

25	$C_6H_{12}O_7$	Gluconic Acid	17.56	195.051	1300000	3900	2800
26	$C_{15}H_{16}N_2O_6$	Propanedioic Acid	10.82	319.0938	480000	480000	3200
27	$C_8H_{15}NO_3$	6-Acetamidohexanoic Acid	13.75	172.0979	33000	4300	3000
28	$C_4H_6O_5$	Malic Acid	1.25	133.0142	-	460000	-
29	$C_6H_6O_6$	Trans-Aconitic Acid	17.27	173.0096	-	220000	-
30	$C_{13}H_{14}N_2O_2$	Tetrahydroharman-3-Carboxylic Acid	7.74	229.0985	1700	460	-
31	$C_{11}H_{11}NO_3$	Indolelactic Acid	15.9	204.0668	2100000	5500	3100
32	$C_6H_8O_7$	Citric Acid	3.28	191.0201	3900000	120000	2000
33	$C_{29}H_{44}O_6$	Polygalic Acid	18.03	489.2078	3200000	2200000	4000
34	$C_{18}H_{32}O_5$	Corchorifatty Acid F	20.07	327.2177	16000	1400000	-
35	$C_{16}H_{11}NO_7$	4-Nitro-5-Phenacylphthalic Acid	1.98	328.0456	1000000	-	850
36	$C_6H_{10}O_8$	Saccharolactic Acid	1.11	209.0307	63000	120000	8300
37	$C_{14}H_{16}N_2O_5$	(2S,4S)-Monatin	7.21	291.0995	89000	430	-
38	$C_{14}H_{18}N_2O_5$	Aspartame	8.45	293.1148	15000	3000	-
39	$C_{15}H_{10}O_4$	Daidzein	18.58	253.051	-	690000	-
40	$C_{17}H_{15}NO_3$	Indoprofen	16.61	280.0982	630000	550000	330
41	$C_9H_{17}NO_5$	Vitamin B5	3.92	218.104	63000	94000	600
42	$C_{14}H_8N_4O_4$	5-Azidoalrestatin	17.3	295.0471	34000	220000	-
43	$C_{13}H_{25}NO_3$	N-Undecanoylglycine	20	242.1762	1000000	990000	-
44	$C_{19}H_{33}N_5O_7$	p-Coumaric Acid	4.82	442.2311	7200	-	-
45	$C_9H_8O_3$	2-Hydroxycinnamic Acid	6.32	163.0403	20000	19000	220
46	$C_7H_6O_4$	2,5-Dihydroxybenzoic Acid	7.92	153.0197	260000	6200	780
47	$C_{30}H_{32}O_{10}$	Trivaric Acid	19.23	551.1921	350000	550000	13000
48	$C_{10}H_{15}N_3O_5$	Benserazide	1.3	256.094	200000	13000	5000
49	$C_{12}H_{12}N_2O_2$	3,3'-Dihydroxybenzidine	6.83	215.0829	29000	4100	3700
50	$C_6H_{12}O_6$	Glucose	10.6	179.0564	760000	2000000	70000
51	$C_{10}H_{12}O_4$	Acetosyringone	15.11	195.0667	260000	2400	7500
52	$C_{16}H_{12}O_6$	Diosmetin	19.76	299.0564	550000	120000	9600

53	$C_5H_{12}N_8S$	Ethyl 4-hydroxybenzoate	13.43	215.0834	13000	1800	-
54	$C_{17}H_{15}NO_4$	Eutropoflavin	15.59	296.0931	340000	190000	560
55	$C_{23}H_{32}N_4O_8$	Caspase-1 Inhibitor I	16.83	491.215	37000	-	4000
56	$C_{17}H_{17}NO_4$	N-Caffeoyltyramine	17.35	298.1075	28000000	1300000	20000
57	$C_{28}H_{31}NO_8$	Hygrocin A	17.9	508.1974	1600000	180000	50000
58	$C_{29}H_{31}NO_8$	Desacetylravidomycin	18.6	520.1975	340000	56000	9300
59	$C_{18}H_{32}O_5$	9,12,13-trihydroxy-10,15-octadecadienoic acid	17.02	328.0211	1100	410000	900
60	$C_{34}H_{32}N_2O_8$	Cannabisin B	18.86	595.2082	5700	2900000	-
61	$C_{34}H_{30}N_8O_6$	1,3-Benzenediol	19.44	645.2218	1400000	360000	67000
62	$C_{10}H_{14}N_2O_4$	Carbidopa	3.3	225.0877	-	3500	-
63	$C_{14}H_{23}N_3O_6$	Valclavam	4.71	328.1514	10000	-	8600
64	$C_{10}H_9N_7O$	Furterene	1.02	242.0801	720000	290000	720
65	$C_{17}H_{17}NO_4$	N-[(diphenylmethoxy)acetyl]-glycine	1.28	176.9361	43000	210000	4000
66	$C_{12}H_{24}N_2S_2$	N,N'-Dipentylethanedithioamide	1.32	259.1306	14000	310	-
67	$C_{29}H_{41}N_5O_9$	Tigecycline Hydrate	11.48	602.2831	-	2200	-
68	$C_{11}H_{18}N_2O_4$	3'-Hydroxyamobarbital	12.64	241.1198	470000	-	6900
69	$C_{17}H_{23}N_7O$	Phidianidine B	16.71	340.1885	87000	-	500
70	$C_{18}H_{19}NO_4$	Feruloyltyramine	18.09	312.1235	14000000	6700000	73000
71	$C_{22}H_{24}N_2O_4$	Bis-Coumaramidobutane	18.29	379.1664	260000	380000	-
72	$C_{11}H_{10}N_6$	Bentemazole	3.42	225.0885	120000	-	770
73	$C_{21}H_{17}NO_5$	Dihydrochelirubine	19.98	362.1032	1300000	40000	2900
74	$C_{25}H_{34}N_4O_8$	Cinitapride	14.83	517.2299	3600	-	300
75	$C_{12}H_{15}NO_4$	3-Hydroxycarbofuran	17.07	236.0933	66000	1000	3500
76	$C_7H_2N_4O_2$	3-Nitropyridine-2,4-Dicarbonitrile	17.28	173.0107	38000	220000	-
77	$C_{30}H_{55}N_5O_5$	Clavatustide A	17.61	564.4125	340000	-	2500
78	$C_{28}H_{29}NO_7$	Desmethylrocaglamide	18.67	490.1869	1200000	470000	2100

79	$C_{26}H_{43}N_5O_7$	Des(Benzylpyridyl) Atazanavir	18.72	536.309	21000	-	840
80	$C_2H_2N_2O_8$	1,2-Bis(Nitrooxy)-1,2-Ethanedione	19.41	180.9733	89000	36000	3000
81	$C_{25}H_{14}N_2$	[(Coronen-1-yl) methylidene] Hydrazine	1.06	341.1092	120000	16000000	300
82	$C_4H_8N_2O_6S$	N-Hydroxysulfosuccinamide	2.53	211.002	410000	-	280
83	$C_{14}H_{16}N_2O_3$	Phetharbital	7.52	259.1091	22000	-	16000
84	$C_{18}H_{28}O_9$	Tuberonic Acid Glucoside	13.87	387.1666	230000	-	39000
85	$C_{14}H_{16}N_2O_4$	5-Benzylacyclouridine	15.65	275.1045	190000	19000	11000
86	$C_9H_{10}O_4$	Homovanillic acid	13.05	301.1089	220000	7600	590
87	$C_{17}H_{13}N_7$	Aplidiopsamine A	16.35	314.1153	150000	-	1400
88	$C_7H_{10}N_6O_2$	8-Hydrazinotheophylline	18.12	209.0802	490000	480000	2200
89	$C_{20}H_{17}NS$	2-(1-Phenylethyl)-10H-Phenothiazine	1.00	302.1017	-	150000	-
90	$C_6H_2N_4$	Pyrazine-2,3-Dicarbonitrile	3.69	129.0204	-	730	-
91	$C_5H_{10}O_3$	2-Methoxyethyl Acetate	4.96	117.0559	370000	770	9300
92	$C_9H_{16}N_2O_2$	Apronal	5.61	183.1143	61000	-	7000
93	$C_9H_{17}NO_4$	Acetylcarnitine	1.68	202.1088	510	950	-
94	$C_{15}H_{23}N_3O_5$	Streptcytosine F	8.37	324.1557	4700	-	-
95	$C_{18}H_{19}NO_5$	Cantharidin	17.04	328.1193	380000	380000	5800
96	$C_{17}H_{17}NO_3$	Morphinone	17.94	282.1132	5300000	4600000	68000
97	$C_{15}H_{10}O_6$	Fisetin	19.16	285.0404	1900000	210000	82000
98	$C_5H_5N_5O$	Guanine	3.25	150.0423	760000	-	400
99	$C_{14}H_{17}N_5O_8$	Succinyladenosine	6.18	382.1008	58000	39000	93000
100	$C_{10}H_{13}N_5O$	Isopentenyladenine	3.93	218.1041	160000	390	3000
101	$C_9H_{12}N_2O_6$	Uracil Riboside	1.31	243.0628	210000	-	5500
102	$C_6H_{12}O_3$	2-Ethoxyethyl Acetate	11.12	131.0716	4200	320000	-
103	$C_{15}H_{22}O_5$	Artemisinin	13.94	281.1399	360000	190000	-
104	$C_9H_{10}O_3$	Ethylparaben	14.34	165.0559	33000	-	77000
105	$C_{20}H_{27}N_3O_6$	Phebarbamate	16.08	404.1832	46000	-	5900

106	$C_{20}H_{38}O_7S$	Docusate Hydrogen	19.01	421.2266	53000	22000	-
107	$C_{29}H_{28}O_9$	Schisantherin D	19.3	519.1658	44000	430000	-

FHS, *L. plantarum* fermented hemp seeds; RHS, raw (unfermented) hemp seeds.
